# RETRACTION: Tamarind: A diet‐based strategy against lifestyle maladies

**DOI:** 10.1002/fsn3.4091

**Published:** 2024-04-05

## Abstract

**RETRACTION:** Muhammad Sajid Arshad, Muhammad Imran, Aftab Ahmed, Muhammad Sohaib, Azmat Ullah, Mehr un Nisa, Gule Hina, Waseem Khalid, and Hafiza Rehana. “Tamarind: A Diet‐Based Strategy against Lifestyle Maladies,” *Food Science & Nutrition* 7 (2019): 3378‐3390, https://doi.org/10.1002/fsn3.1218.

The above article, published online on 27 September 2019 in Wiley Online Library (wileyonlinelibrary.com), has been retracted by agreement between the journal's Editor‐in‐Chief, Y. Martin Lo, and Wiley Periodicals, LLC. The retraction has been agreed to following concerns about the peer review process. The editorial office found unambiguous evidence that the manuscript was accepted solely based on compromised and insufficient reviewer reports. This evidence was further investigated and verified by the publisher and the Editor‐in‐Chief. As a result, the conclusions of this article are not considered reliable. The authors were notified of the retraction and disagreed with this decision.

**RETRACTION:** Muhammad Sajid Arshad, Muhammad Imran, Aftab Ahmed, Muhammad Sohaib, Azmat Ullah, Mehr un Nisa, Gule Hina, Waseem Khalid, and Hafiza Rehana. “Tamarind: A Diet‐Based Strategy against Lifestyle Maladies,” *Food Science & Nutrition* 7 (2019): 3378‐3390, https://doi.org/10.1002/fsn3.1218.

The above article, published online on 27 September 2019 in Wiley Online Library (wileyonlinelibrary.com), has been retracted by agreement between the journal's Editor‐in‐Chief, Y. Martin Lo, and Wiley Periodicals, LLC. The retraction has been agreed to following concerns about the peer review process. The editorial office found unambiguous evidence that the manuscript was accepted solely based on compromised and insufficient reviewer reports. This evidence was further investigated and verified by the publisher and the Editor‐in‐Chief. As a result, the conclusions of this article are not considered reliable. The authors were notified of the retraction and disagreed with this decision.

